# The effects of temperature on the proxies of visual detection of *Danio rerio* larvae: observations from the optic tectum

**DOI:** 10.1242/bio.047779

**Published:** 2020-07-21

**Authors:** Ewa Babkiewicz, Michał Bazała, Paulina Urban, Piotr Maszczyk, Magdalena Markowska, Z. Maciej Gliwicz

**Affiliations:** 1 Department of Hydrobiology, Faculty of Biology, University of Warsaw at Biological and Chemical Research Centre, Żwirki i Wigury 101, 02-089 Warsaw, Poland; 2 Laboratory of Neurodegeneration, International Institute of Molecular and Cell Biology in Warsaw, Księcia Trojdena 4, 02-109 Warsaw, Poland; 3 Laboratory of Functional and Structural Genomics, Centre of New Technologies, University of Warsaw, Banacha 2c, 02-097 Warsaw, Poland; 4 College of Inter-Faculty Individual Studies in Mathematics and Natural Sciences, University of Warsaw, Banacha 2c, 02-097 Warsaw, Poland; 5 Department of Animal Physiology, Faculty of Biology, University of Warsaw, Miecznikowa 1, 02-096 Warsaw, Poland

**Keywords:** Detection distance, Reaction distance, Perception, Zebrafish, Temperature, Visual stimuli

## Abstract

Numerous studies have indicated that temperature improves the visual capabilities of different ectotherms, including a variety of fish species. However, none of these studies has directly tested whether elevated temperature extends the visual detection distance – the distance from which a visual stimulus is detected. To test this hypothesis, we investigated the effect of temperature on the visual detection distance of zebrafish (*Danio rerio*) larvae by measuring the largest distance from a moving target that induced a neural response in the optic tectum. We applied advanced methods of functional calcium imaging such as selective plane illumination microscopy in combination with a miniature OLED screen. The screen displayed an artificial, mobile prey, appearing in the visual field of the larvae. We performed experiments in three temperature treatments (18, 23 and 28°C) on transgenic fish expressing a fluorescent probe (GCaMP5G) that changes intensity in response to altered Ca^2+^ concentrations in the nerves in the optic tectum. Based on the obtained data, we also measured three additional parameters of the neural response in the optic tectum, each being a proxy of sensitivity to changes in the stimulus movement. We did not confirm our hypothesis, since the visual detection distance shortened as the temperature increased. Moreover, all of the three additional parameters indicated a negative effect of the temperature on the speed of the neural response to the stimuli. However, the obtained results could be explained not only by worse visual capabilities at the elevated temperature, but also by the differences in the visual field and in turn, the retinotopic location of the visual stimulus between the temperature treatments, since the stimulus in the experiments moved horizontally rather than forward and backward from the fish's eye.

## Abstract (precis)

**Summary:** During the assessment of the visual detection distance of transgenic *Danio rerio* larvae in response to a visual stimulus using selective plane illumination microscopy, we found that the distance is shorter at elevated temperatures.

## INTRODUCTION

Since temperature affects metabolic (Gilloly et al., 2001) and behavioural (e.g. Chen et al., 2003) as well as almost all other biological rates (Brown et al., 2004), it strongly affects the fitness of ectotherms. The literature provides numerous examples of studies indicating that temperature affects a variety of physiological performances, including the functioning of visual capabilities in terrestrial and aquatic ectotherms. The evidence comes from three groups of studies.

The first group of studies described the observation that several large marine predatory fishes, including several species of swordfishes (Carey, 1982; Fritsches et al., 2005), lamnid sharks (Block and Finnerty, 1994), tunas (Dickson et al., 2000; Sepulveda et al., 2007) and mackerel (*Gasterochisma melampus*, Sepulveda et al., 2007) independently evolved the mechanisms of partial cranial endothermy, or even whole-body endothermy, as in the case of opah (*Lampris guttatus*, Wegner et al., 2015). Opah produces heat through the constant movement of its pectoral fins and minimises heat loss through a series of counter-current heat exchangers within its gills (Wegner et al., 2015). In the case of partial endothermy, as, for example, in the generally ectothermic tunas (family *Scombridae*), cranial counter-current heat exchangers, retia mirabilia, conserve metabolic heat, allowing cranial temperatures to be elevated above the ambient water temperature (Holtz, 2013). These abilities allow elevated eye and brain temperatures to be maintained (Block, 1986; Sepulveda et al., 2007; Fritsches et al., 2005; Runcie et al., 2009). Despite their high energy costs, these phenomena are treated as an adaptation improving the ability to detect and in turn to hunt prey more efficiently while diving in deep and cold marine water layers (Block and Finnerty, 1994). In the case of swordfishes, partial cranial endothermy helps to prevent the rapid deterioration of temporal resolution with decreasing temperature when the swordfish descends to deeper and cooler water layers (Fritsches et al., 2005). The whole-body endothermy of opah allows for enhanced temporal resolution and neural conductance of the eye and brain (Wegner et al., 2015).

The second group of studies assessed the effect of temperature on the activity of photoreceptors in the retina, mainly of insects and fishes in response to light stimuli, most often using the electroretinography method. Some of these studies tested the effect of temperature on the spectral sensitivity of retinal photoreceptors to different wavelengths of light in the visible range. For instance, Saszik and Bilotta (1999) performed experiments in the same intensity and frequency, but with different spectral compositions of light to assess the spectral sensitivity of adult zebrafish (*Danio rerio*) reared in darkness and in different temperatures before the experiments. They revealed that individuals housed in the warmer temperature had greater sensitivity to longer wavelengths. In this way, they indirectly showed that the retina of fish reared in warmer temperatures had a greater contribution of rhodopsin in relation to porphyropsin, since the latter photopigment absorbs longer wavelengths. The results are consistent with previous studies (Bridges, 1964; Allen and McFarland, 1973) that examined the rod pigment composition of the golden shiner (*Nofemigonus crysoleticas*) during different seasons and found that the amount of porphyropsin in fish caught during winter was greater than in individuals caught during summer. Numerous studies also assessed the effect of temperature on the speed and accuracy of the response, measuring the retinal response to light stimuli of the same spectral composition but at different illumination frequencies using temporal resolution as a measurement of the ability to handle different rates of change in luminescence. These studies generally revealed that a higher temperature increases the speed and improves the accuracy (precision) of the response, which would contribute to the detection of smaller and/or faster moving objects (e.g. the prey or the predator). This was shown, for instance, for the blowfly (*Calliphora vicina*; Tatler et al., 2000), the predatory owlfly (*Libelloides macaronius*; Belušič et al., 2007), and a variety of fish species, including European eel (*Anguilla anguilla*), Prussian carp (*Carassius gibelio*), spotted dogfish shark (*Scyliorhinus canicula*; Gačić et al., 2015), and swordfish (*Xiphias gladius*; Fritsches et al., 2005). It was also revealed that temperature increases the frequency of responses of the ocellar neurons in the retina to the third-order neurons in the protocerebrum of the desert locust (*Schistocerca gregaria*; Simmons, 2011).

The third group of studies assessed the effect of temperature on the reaction distance to moving objects, including prey, of some species of frogs and fish, as a behavioural proxy for visual prey detection, (e.g. Aho et al., 1993; Bianco et al., 2011; Gliwicz et al., 2018). For instance, it was recently revealed that the reaction distance toward planktonic prey of two juvenile planktivorous fishes, rudd (*Scardinius erythrophthalmus*) and Malabar danio (*Devario malabaricus*), is greater at higher than at lower temperatures (Gliwicz et al., 2018). However, the reaction distance is not a perfect proxy for visual prey detection, since this distance not only depends on visual abilities, but also on the intention of the individual to react or not, to the encountered object (Abram et al., 2017). Therefore, a greater reaction distance to prey at a higher temperature may not only be due to greater detection distance, but also to the greater motivation of the fish to attack the prey from a greater distance at a higher temperature.

Most of the aforementioned studies suggested or even indicated positive effects of temperature on the visual capabilities apparent in changes in different physiological and optical parameters. However, none of them directly tested whether an elevated temperature causes an extension of the visual detection distance, defined as the distance from which the visual stimuli are perceived. Although the thermal sensitivity of detection distance has not been assessed yet, several studies assumed that the detection distance is independent of temperature (Dell et al., 2014; Pawar et al., 2012).

There are some possible ways to estimate detection distance *in vivo*, either by using the electroretinography method (Chrispell et al., 2015), the electrophysiology of brain regions (Ramdya et al., 2006), functional MRI (Kabli et al., 2006) or by using single plane illumination microscopy – one of the newest functional imaging methods (SPIM; Huisken et al., 2004). The latter method involves the long-term live imaging of medium-sized, intact organisms, such as zebrafish larvae, which for the convenience of observation could express fluorescent protein probes that change fluorescence in response to alterations in the concentration of Ca^2+^, for example in neurons (Ahrens et al., 2013), or possess an exogenous reporter (e.g. Ashworth and Bolsover, 2002).

The advantages of using SPIM and transgenic fish larva provides the possibility to observe an intact organism that changes fluorescence in response to alterations in the concentration of Ca^2+^ in the neurons, allowing the assessment of the detection distance from the visual stimuli on the almost complete visual pathway through the ocular nerves to the optic tectum.

This study aimed to test the hypothesis that an elevated temperature extends the visual detection of visually-oriented zebrafish larvae. The detection distance was assessed using the SPIM method and defined as the largest distance from a moving target appearing in the fish's visual field that induced a neural response in the optic tectum.

## RESULTS

The oxygen concentration in the E3 medium measured before the experiments outside the sample chamber of the Lightsheet Z.1 microscope differed in different temperatures (10.6±0.2, 9.6±0.2 and 8.4±0.2 mg l^−1^ for 18, 23 and 28°C, respectively). However, the oxygen concentration in the chamber during the experiments was very similar in each of the temperature treatments (9.5±0.3). The oxygen concentration inside the glass capillary filled with agarose and submerged in the E3 medium was only slightly lower (±0.2–0.3 mg l^−1^) than directly in the E3 medium in each of the three experimental temperatures. The reported range of oxygen concentrations was relatively high in comparison with the oxygen concentrations reported in the wild (ranged from 1.2 to 20.0 mg l^−1^) (Khan et al., 1970) and similar to the oxygen concentrations noted in the larvae's home tank (∼8 mg l^−1^ at 28°C).

**Fig. 1.**
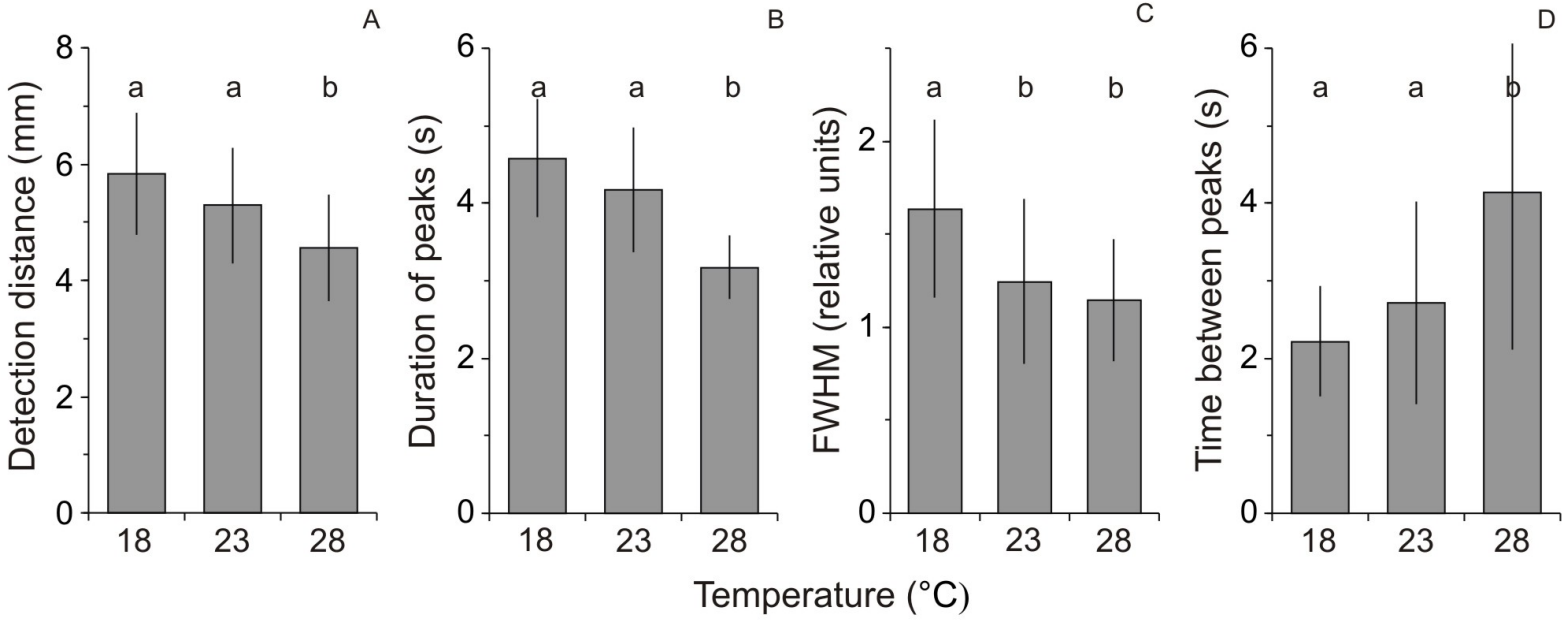
**The experimental results.** (A) Detection distance (mean+1s.d.), (B) duration of peaks (mean+1s.d.), (C) full width at half maxima of peaks (FWHM, mean+1s.d.), and (D) the time between two adjacent peaks (mean+1s.d.) assessed for the peaks of relative florescence reflecting neural activity in a region of the OT in response to the visual stimuli at three temperature treatments. Results that share the same letter do not differ significantly from each other.

Statistical analysis showed that the detection distance, duration of peaks, FWHM and time between two adjacent peaks differed significantly between temperatures (Table 1). The detection distance was shorter at the higher temperature, with a significant difference found between 23 and 28°C (*P*=0.022, Scheffe's post hoc) and between 18 and 28°C (*P*<0.0001, Scheffe's post hoc; Fig. 1A). Results observed at 18 and 23°C did not differ significantly (*P*=0.110; Fig. 1A). The duration of peaks was lower at the higher temperature, with a significant difference found between 23 and 28°C (*P*<0.0001, Tamhane's post hoc) and between 18 and 28°C (*P*<0.0001, Tamhane's post hoc; Fig. 1B). Results observed at 18 and 23°C did not differ significantly (*P*=0.103; Fig. 1B). The FWHM was lower at the higher temperature, with a significant difference found between 18 and 23°C (*P*<0.0001, Scheffe's test post hoc) and between 18 and 28°C (*P*<0.0001, Scheffe's test post hoc; Fig. 1C). Results observed at 23 and 28°C did not differ significantly (*P*=0.623; Fig. 1C). The time between two adjacent peaks was greater at the higher temperature, with a significant difference found between 23 and 28°C (*P*<0.0001, post-hoc test) and between 18 and 28°C (*P*<0.0001, Dunn's post-hoc test; Fig. 1D). Results observed at 18 and 23°C did not differ significantly (*P*=0.170; Fig. 1B).

**Table 1.**
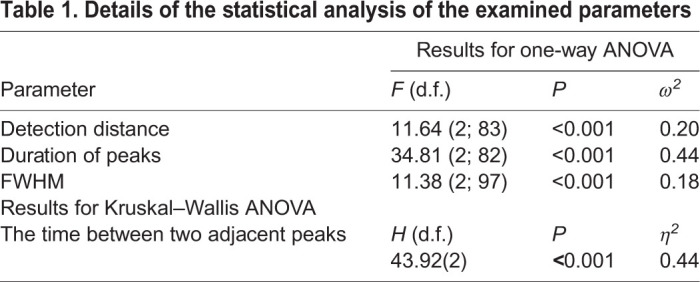
**Details of the statistical analysis of the examined parameters**

## DISCUSSION

The results of our study did not confirm the hypothesis that an elevated temperature extends the visual detection distance of visually oriented zebrafish larvae, since the higher temperature resulted in a decrease rather than increase of this parameter. Moreover, the other measured parameters indicated a negative effect of temperature on the speed of the neural response to the stimuli of the larvae (e.g. shorter duration of peaks at elevated temperature). Therefore, the results contradict the majority of studies that revealed a positive rather than negative effect of temperature on a variety of physiological (optical and neurological) parameters describing the visual capabilities of different species of ectotherms, including fishes (Tatler et al., 2000; Belušič et al., 2007; Gačić et al., 2015). We propose at least four potential explanations for the opposite tendency of the temperature effects obtained in the majority of earlier physiological and behavioural studies on visual abilities and in this study.

The first explanation is that reduced detection ability could be due to increased receptor and synaptic noise at higher temperatures (a noise arising from the randomly occurring thermal isomerisation of the retinal photoreceptors; Aho et al., 1988), even though simultaneously, higher temperatures would positively improve other aspects of visual performance such as temporal resolution. This would reflect the classic trade-off in visual processing – improved temporal resolution achieved at the cost of sensitivity, in this case in the form of a loss in the detectability of an approaching object. Greater levels of neural noise will directly impact the signal-to-noise ratio of a visual signal, and this in turn impacts the minimum contrast of objects that can be detected. This potentially could also be visible in the decreasing ratio of the maximal to the basal fluorescence of the peaks with increasing temperature. Since, particularly in aquatic environments, the contrast of objects rapidly declines with distance (due to light scattering from the particles suspended in the water column, Agrawal et al., 2008), only slight increase in noise could significantly shorten detection distance. This is confirmed by the fact that the main reason why typical prey animals in aquatic environments attempt, by every means possible (such as transparency; McFall-Ngai, 1990; Johnsen, 2001), to reduce their visual contrast against the background.

The second most likely explanation for the negative effect of higher temperature on the measured parameters are oxygen deficiencies in the brain due to the elevated metabolic rate (Clarke and Johnston, 1999; Killen et al., 2010), especially as the functioning of the sensory systems and brain signalling are energy demanding processes (Laughlin et al., 1998; Laughlin, 2001). We cannot exclude the possibility that the oxygen demands at the highest temperature were greater than the experimental environment could provide. However, the concentration of oxygen in the E3 medium inside sample chamber was above 9.0 mg l^−1^ at the highest temperature. This value is rather high compared to the oxygen concentrations in the natural environment or in the larva's home tank (see Results section). Moreover, the threshold that would be harmful for the functioning of the OT of zebrafish larvae should be at a much lower concentration, as zebrafish larvae are known to be significantly resistant to oxygen deficiency, even at high temperatures (Strecker et al., 2011; Mendelsohn, et al., 2008; Pan and Hunt von Herbing, 2017). For instance, it was shown that 5 days post fertilization (dpf) larvae contain various types of globins responsible for oxygen transport and storage, including haemoglobin in the erythrocytes and neuroglobin in the nervous system, more in relation to their body mass compared to older individuals. The rapid expression of neuroglobin is observed from 18 hours post-fertilisation (hpf) to 6 dpf, which is associated with the great number of neurons that are formed during this period in the brain (Tiedke et al., 2011). It was also shown that small-bodied ectothermic individuals (such as larvae) are less susceptible to thermal stress than large-bodied ones because they are able to deliver (relative to their body mass) more oxygen to tissues due to shorter oxygen diffusion distances (Atkinson et al., 2006; Czarnoleski et al., 2017). It was also shown that the larval stages of numerous fish species (including zebrafish) are able to efficiently exchange gas not only thorough the gills but also through all of the skin (Rombough, 1988, 2002).

The other most likely explanation is the difference in the sizes and ontogenetic stages of the ectothermic animals used in the earlier studies and in our study. Contrary to previous studies conducted on adult fish, amphibians or insects, we used small fish larvae (1.2 mm, 5 dpf). Larval stages do not have a fully developed vision system and brain. This would result in an increased sensitivity of visual abilities to a higher temperature and to its rapid changes. The immaturity of the visual system could be seen in different features of the anatomical (cellular) structure of the retina and brain or in their not fully developed functions (Billota and Saszik, 2001; Niell and Smith, 2005). For instance, among the two photoreceptors (rods and cones) present in the retina of adult zebrafish, larval stages (until 15–40 dpf; Billota and Saszik, 2001) only have developed cones, which are responsible for colour vision. Cones would be more sensitive to metabolically related oxygen deficiencies, which is consistent with earlier studies on hypoxia and colour vision capabilities in humans, indicating that hypoxia could impair colour vision (Vingrys and Garner, 1987; Barbur and Connolly, 2011; Hovis et al., 2012). The visual neural circuits in the zebrafish larval brain undergo dynamic formation during its early ontogenesis (Dunn et al., 2016) and temperature also influences neuronal circuits functioning by inducing various physiological processes, such as compensation, tuning and tolerance, making it difficult to precisely predict the overall influence on the output (Robertson and Money, 2012). However, the maturation stage and functioning of the visual system in 5 dpf zebrafish larvae is still advanced enough to allow for the active visual searching and hunting for protozoan prey (Bianco et al., 2011).

The next explanation could be the specificity of the experimental set-up used in our study. The first possible error may be due to the fact that in the experiment, the visual stimulus was moving horizontally across an OLED screen oriented parallel rather than forward and backward to the fish's eye. Therefore, the stimulus was not only moving in the distance from the eye, but was also changing its retinotopic location. As different regions of the zebrafish's retina have different contrast sensitivities, spectral sensitivities and visual acuity (e.g. Zimmermann et al., 2018), the possibility cannot be excluded that the obtained results could be explained not only by worse visual capabilities at the elevated temperature, but also by the differences in the visual field and in turn, the retinotopic location of the visual stimulus between the temperature treatments. Another potential error may be the use of larvae covered with 1.5% low-melting agarose in the experiments, which could result in a decline in oxygen diffusion through the skin and gills, limiting the oxygen supply to the body tissues. However, mounting zebrafish larvae in agarose for long-term light sheet microscopy is a standard method and is claimed to be appropriate for durable (up to 2 h) imaging before it starts to significantly affect the physiology of the larvae (Weber et al., 2014). Moreover, our control measurements indicated that the effect of the agarose covering the larvae inside the microscope sample chamber on the oxygen concentration available to the larvae was negligible. The other potential error could have been a too short acclimation time of the larvae to the given temperature before and during the experiments, even if the highest applied temperature (28°C) was close to the optimal temperature for zebrafish larvae development, which is around 27°C (e.g. Engeszer et al., 2007; Lawrence, 2007; Spence et al., 2008). Even if the oxygen concentration was high in the surrounding environment, the larva's metabolism may have not kept up with the relatively fast (1°C in 3 min) temperature changes. Finally, the last potential methodological error would be using a blue OLED background instead of a white one, which would activate all cone photoreceptors as opposed to the background used, which targets mainly blue cones. However, it should be pointed out that the blue cones are distributed evenly in a mosaic pattern throughout the retina of larval zebrafish (12 hpf–3-4 dpf, Allison et al., 2010; Raymond et al., 1995), therefore, it could be expected that despite small differences of the position of the experimental fish, the OLED display should activate the blue cones to the same extent regardless of the retinotopic location of stimulus perception.

The most interesting interpretation of the opposite tendency of the temperature effects obtained in earlier studies on visual abilities and our study is that the reduced reaction distance at the lower temperature observed in several behavioural studies (Aho et al., 1993; Gliwicz et al., 2018) could be consistent with a shorter detection distance. This may be so because at the lower temperature, the fish could see the prey at a greater distance but not have always attacked when the potential prey was quite far away from the fish. This may be due to the fact that at a low temperature, it is not profitable for fish to catch prey from a great distance since the energy invested to capture it outweighs the energy gained from its consumption (e.g. Werner and Hall, 1974; Griffiths, 1980; Mittelbach, 1983). Although physiological studies showed positive rather than negative effects of temperature on visual acuity, they assessed the effect of temperature on other parameters, including temporal resolution, speed, accuracy or the strength of the retinal response (Tatler et al., 2000; Belušič et al., 2007; Gačić et al., 2015), rather than on the visual detection distance, by observing the brain's response to the visual stimulus. It cannot be excluded that the positive effect of temperature on the retinal response is consistent with its negative effect on the detection distance.

In conclusion, our results seems to be the first ones that assessed the thermal sensitivity of the visual detection distance in zebrafish larvae in response to a moving artificial visual stimulus, indicating the negative effect of temperature on the visual detection distance. Further research is needed to elucidate the physiological mechanisms of this phenomenon. The results may be interesting in the context of previous and future studies with zebrafish as a simple, vertebrate model organism, widely used in biological and medical studies (Bilotta and Saszik, 2001). The visual system of zebrafish has been examined in many previous studies concerning a variety of diseases, including the biology of visual disorders (Goldsmith and Harris, 2003) and human ocular disorders (Gestri et al., 2012). Moreover, of interest are the ecological consequences of the reduced detection distance in zebrafish larvae and how it relates to adult zebrafish, that is, how it possibly changes throughout zebrafish development and ontogenesis.

## MATERIALS AND METHODS

### The approach

**Fig. 2.**
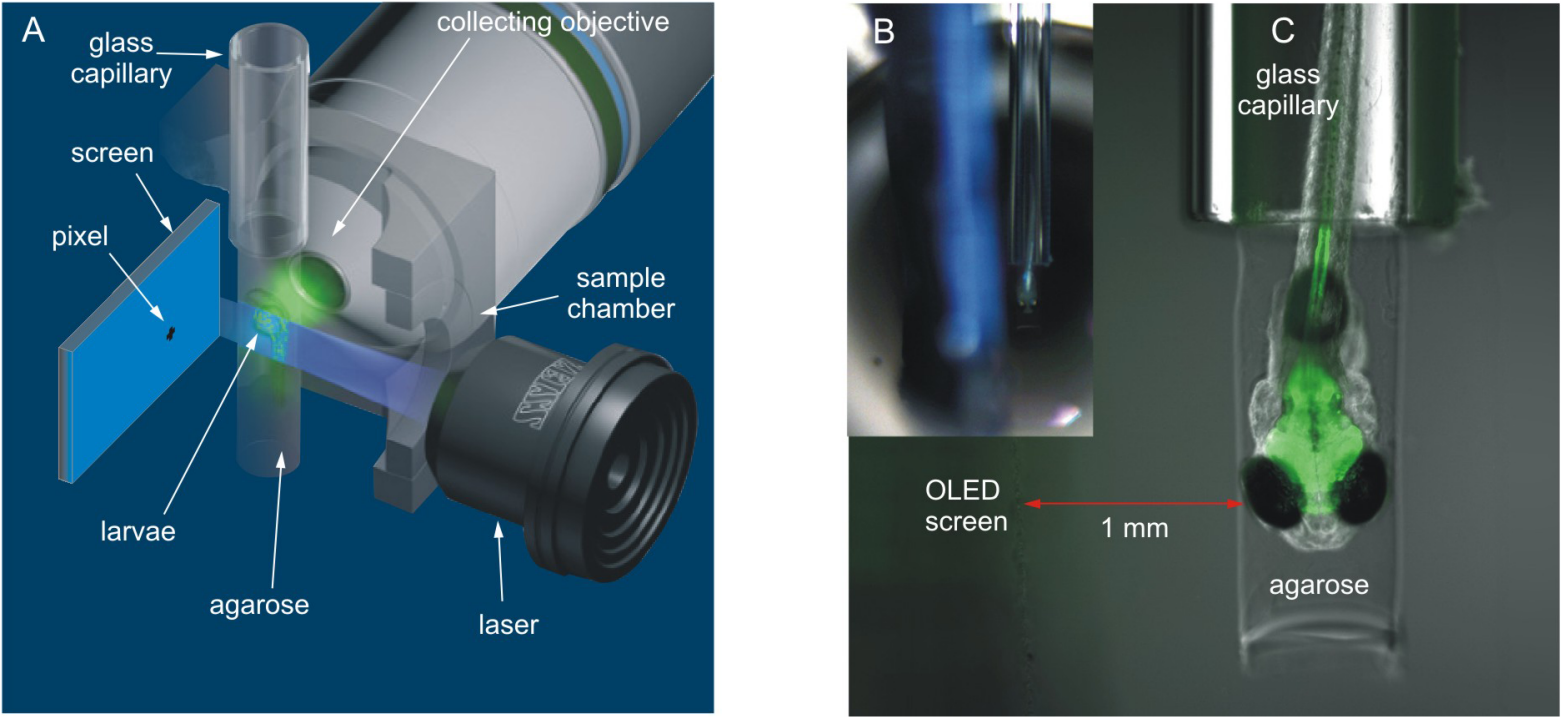
**Position of zebrafish larvae and miniature OLED screen inside the sample chamber of a Lightsheet Z.1 microscope and photographs showing the glass capillary with zebrafish larvae and OLED screen inside the sample chamber of the microscope.** (A) Perspective view (modified picture from the ZEISS Lightsheet Z.1 Light Sheet Fluorescence Microscopy for Multiview Imaging of Large Specimens). (B) The position of the glass capillary with zebrafish larva relative to the screen surface. Photo taken with a camera located on the inside part of the door leading to the sample chamber of the microscope. (C) An enlargement showing larvae illuminated with a laser light.

**Fig. 3.**
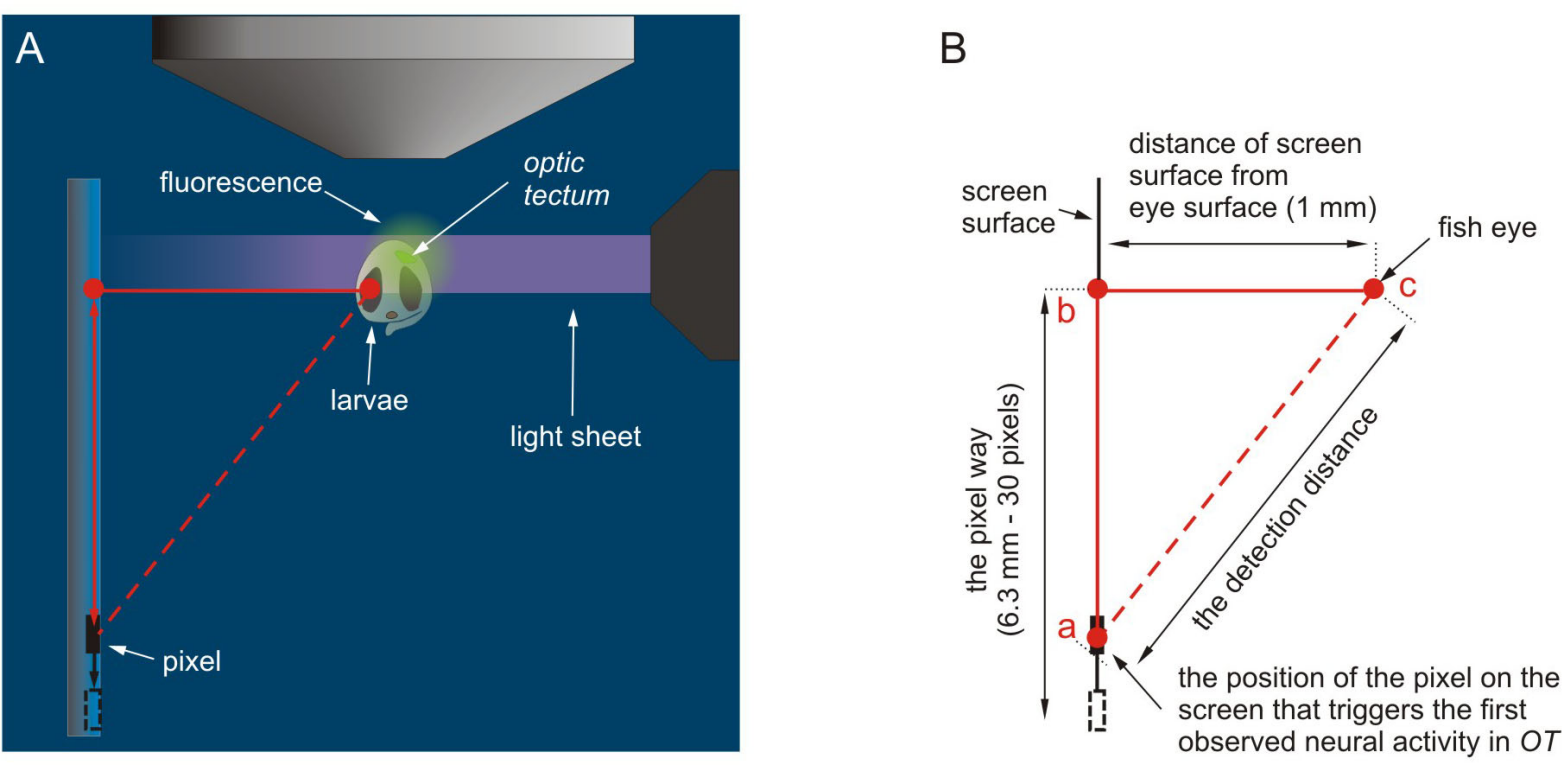
**Pictures showing the zebrafish larva inside the glass capillary and the OLED screen inside the sample chamber of the microscope and the scheme for estimating the detection distance.** (A) Top view. Note that the red triangle represents the relative position of the fish eye in relation to the screen and the position of the pixel on the screen, which induces the first neural signal in the OT (modified picture from the ZEISS Lightsheet Z.1 Light Sheet Fluorescence Microscopy for Multiview Imaging of Large Specimens). (B) A detailed scheme for designating the detection distance using the Pythagorean theorem. The red dotted line represents the length assumed to be the detection threshold distance.

**Fig. 4.**
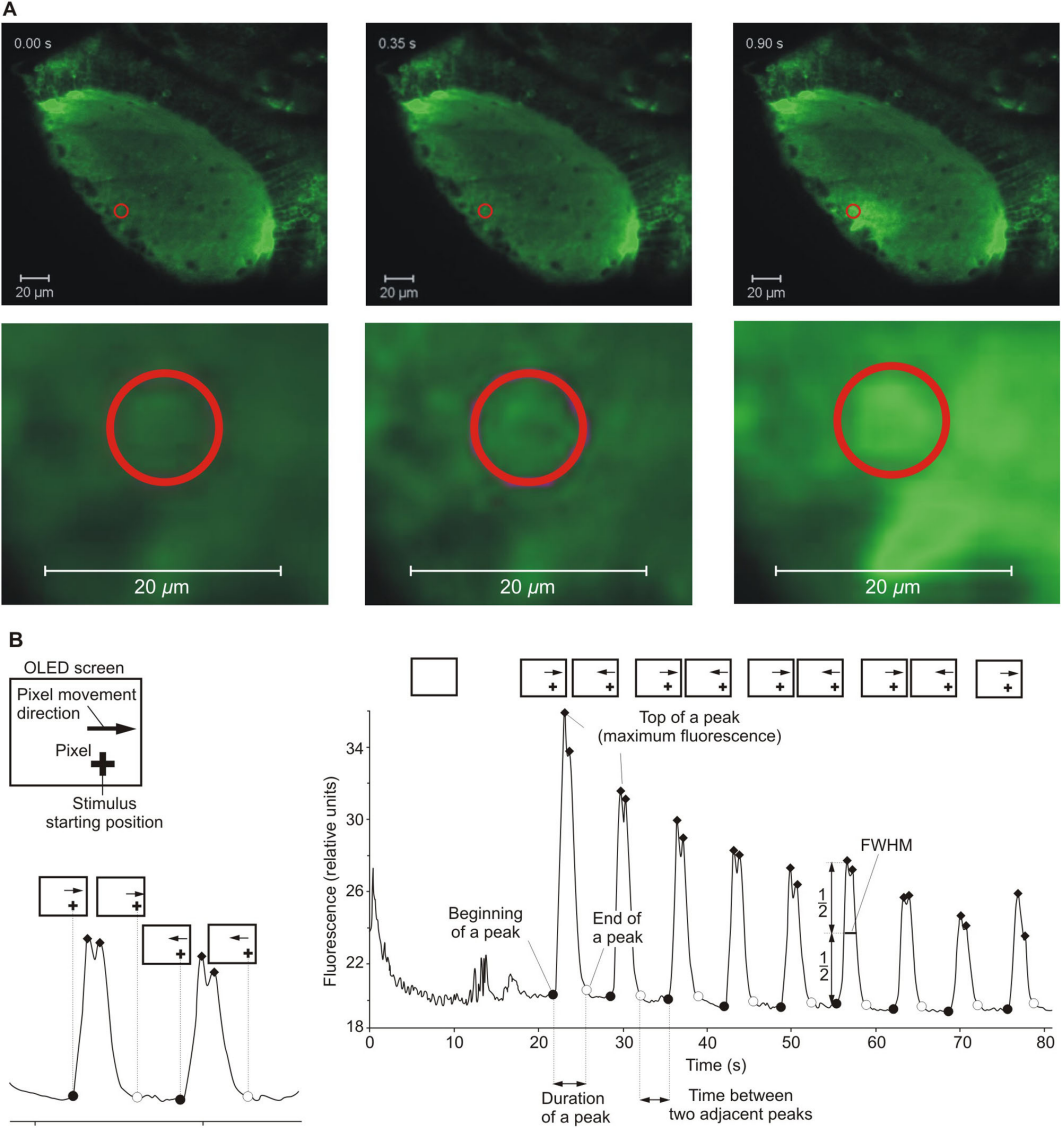
**Details of data analysis.** (A) Experimental time-lapsed images (without enlargement in the upper panel and with enlargement in the lower panel) analysed in ZEN software, with the red ring marking the ROI. Note that at starting, the time-point intensity of fluorescence was at the basal level and increased as the visual stimulus approached and moved opposite to the fish eye. (B) An example of the florescence graph at 23°C with details of the measured parameters on the peaks and the scheme of the correspondence of the position of the visual stimulus on the OLED screen and the peak line.

To test our hypothesis, we performed 20 trials, each with a single *D.* *rerio* larvae (5 dpf) in three temperature treatments (18, 23 and 28°C). We performed the experiments using a Lightsheet Z.1 microscope (ZEISS, Germany), connected to a computer with ZEN software, at the International Institute of Molecular and Cell Biology in Warsaw, Poland. The microscope uses one of the newest microscopy methods, SPIM (Huisken et al., 2004). This method involves long-term live imaging of medium-sized, intact, transgenic organisms, such as fish larvae expressing fluorescent protein probes sensitive to Ca^2+^ concentrations in neurons (Ahrens et al., 2013). This method allowed us to record the first neural signal in the optic tectum (OT), a part of the brain responsible for the processing of visual stimuli. This stimulus was a moving pixel displayed on a miniature OLED screen, located inside the sample chamber of the microscope, simulating an artificial, mobile planktonic animal appearing in the visual field of the larvae (Figs 2 and 3). Using the Lightsheet Z.1 microscope and ZEN software, we obtained data of the fluorescence intensity at a given time-point in a region of the OT of the larvae in response to the visual stimuli. Then, the data were analysed using MatLab and Python software. During the experiments, for each fish, in each temperature treatment, the pixel displayed on the screen moved toward and away from the larvae and appeared in a loop of 20 times in the visual field volume of the fish. This generated ten peaks of neural activity shown as a relative fluorescence intensity in a given time-point (Fig. 4). First, we drew graphs of the fluorescence fluctuations for each temperature treatment. Next, based on the data of neural activity in a given time-point, the distance of the moving object from the fish eye, and the movement speed of the pixel, we calculated the detection distance (DD), assumed as the distance from the stimulus that triggers the first neural response in a region the OT (Fig. 2B). Based on the fluorescence data in each graph, we also measured three additional parameters, each being a proxy of sensitivity to changes in stimulus movement and irrelevant of detection distance: (1) the mean duration of the peaks (i.e. the mean time between the beginning and the end of the peaks appearing in the OT in response to the moving stimulus, describing the time of the stimulus perception), (2) the mean full width at half maxima of the peaks (FWHM, describing the width of the peaks at their half maxima), and (3) the mean time between two adjacent peaks (i.e. the mean time between the end of one peak and the beginning of the next peak appearing in a region of the OT in response to the moving stimulus).

### Experimental animals

Zebrafish larvae (*D.* *rerio*) used in the experiments were the offspring of adult individuals maintained in standard conditions (Matthews et al., 2002). The larvae were kept in standard E3 medium at 28°C in a natural photoperiod (14:10 LD) until 5 dpf. We used a transgenic fish line expressing green fluorescent protein probes sensitive to Ca^2+^ in the neurons [line Tg(HuC:GCaMP5G); Ahrens et al., 2013]. Individuals of this line were also deprived of skin pigment [Nacre (*mitfa*−/−)], which facilitates observations of changes in the fluorescence intensity inside the brain through the transparent skin. All of the three temperatures used in the experiments were within the range of temperatures found in the natural environments of zebrafish (Engeszer et al., 2007; Spence et al., 2008; Lawrence, 2007). After the experiments, the larvae were anaesthetised by overdosing with MS-222 [A5040, Sigma-Aldrich; this procedure does not require permission from the Local Ethics Committee due to Directive 2010/63/EU (EU, 2010) stating that fish depending on the yolk as a source of nutrition are not regarded laboratory animals and have no status as protected organisms].

### Experimental system

We applied the original combination of a miniature OLED screen and the imaging capabilities of the Lightsheet Z.1 microscope. This provided the opportunity to evaluate the largest distance between the fish larvae and the view of artificial planktonic prey that caused a reaction in the neural activity of the brain, in response to the presence of the prey-like object in the visual field. The prey was presented to a zebrafish larvae inserted into a glass capillary in front of a collecting objective, recording the neural signal from the fish's OT (Fig. 2A,B). The sample chamber in the microscope was equipped with a Peltier module, which was precisely cooling or heating the E3 medium inside the chamber. Prey simulation was conducted by using a monochrome OLED display [Sparkfun (USA); #13003; resolution 64×48 pixels; outline dimension 18.46 (W)×18.10 (H)×1.45 (T) mm; visual area 15.42×12.06 mm; active area 13.42 (W)×10.06 (H) mm; dot (pixel) size 0.19×0.19 mm; dot (pixel) pitch 0.21×0.21 mm; contrast ratio 2000:1]. The display was controlled by an Arduino UNO micro-controller (ARDUINO UNO REV3, Arduino.cc). Cables connecting the display to the ARDUINO unit (except for the display surface) were sealed with waterproof polymer glue and a transparent hot melt adhesive. A moving target was displayed as a single, dark object on a light background (with a 2000:1 contrast), shaped as a cross, moving at a constant speed of 2.1 mm s^−1^ (calculated as: length of pixel pitch [0.21 mm]/pixel jump speed [0.1 s]) in a horizontal direction toward and away from the larvae. Its angular speed was equal to 20° s^−1^ (the one-way length of the track was 30 pixels, which equalled 6.3 mm). The cross-like shape was created with 5 pixels, and its outer dimensions were 0.63 mm×0.63 mm (angular size equal to 2.22°). It meets the already observed phenomenon that zebrafish larvae behaviourally react to a view of an object with an angular size of between 1 and 10° (Semmelhack et al., 2014; Del Bene et al., 2010; Bianco et al., 2011). The code for the pixel animation is available online from the Github repository (https://github.com/riverraid1/artificial_prey.git). The intensity of the light provided by the display was equal to 0.014 W m^−2^ measured a by Li-Cor 189 quantum sensor measuring radiance (Li-Cor Biosciences^®^).

### Experimental design

A 5-dpf larva was individually submerged in 1.5% low melting point agarose at 33°C (A9414, Sigma-Aldrich) and drawn into a glass capillary, to mount it in the microscope sample chamber (Fig. 1A). The larva was imaged from the dorsal side of the head and was submerged in the E3 medium from the tip of the head up to the middle of the swimming bladder (Fig. 1B,C). The OLED display was located and fixed on one side of the sample chamber (Fig. 1B). The zebrafish eye surface was 1 mm distant from the nearest point of the display (Figs 1B and 2A,B). After mounting a fish in the microscope sample chamber (filled with E3 medium of 28°C), imaging settings and temperature treatments were adjusted (temperature acclimatization speed was 1°C change up or down in relation to the starting temperature every 3 min). Then, the recording of time lapsed images of the OT region was initialised and we screened the OT region for any spontaneous and basal fluorescence (graphs containing the baseline traces in Fig. S1). Before initiating the pixel movement, the pixel was displayed as a motionless object (outside the visual field of the larvae). The position of the pixel was set in such a way that it took more than 2 s from the start of the pixel moving on the OLED screen toward the fish eye to evoking the first OT response (this is also visible in Movie 1). At the start of the prey simulation, only the motion of the pixel appeared (there was no rapid lighting up of the entire display). Acquisition of the neural signal appearing and disappearing in response to the motion of the pixel in the visual field of the larvae was stopped 30 s after the end of the pixel movement. Approximately 10–30 s after starting the recording, the time-lapsed images of the pixel movement were manually initiated in Arduino UNO and lasted for ten cycles (2 min in total). Imaging parameters were as follows: frame dimensions X:1920, Y:1920; live image size X: 877.42 µm, Y: 877.42 µm; objective Plan-Apochromat 20×/1.0 UV-VIS; zoom 0.5; laser 488 nm at 15% power; exposure time 20.0 ms; time gap between exposures 30 ms; lightsheet thickness 6.40 µm. We found that the signal in the OT was visually synchronised with the movement of the pixel (synchronization of the recordings of the OT signal with a pixel movement could be seen on the video of the experimental set-up – Movie 1). Each fish was started at the different temperature (they were screened in a sequence). However, to avoid artefacts from the rapid temperature changes, we always transferred the fish from 28°C to 28°C and then acclimatised the fish to the desired temperature. The rate of temperature change was 1°C down or up in relation to the starting temperature, every 3 min. The first fish was put into 28°C, screening of a region in the OT was conducted, then a slow acclimatization (over 15 min) to 23°C was done and a second screening was conducted, then the next acclimatization (again over 15 min) was done to 18°C, and then the third screening was taken. The second fish, however, started as usual at 28°C, and then was slowly acclimatised (over 30 min) to 18°C, the screening was conducted, and then the next two screenings were conducted at 23°C and 28°C, respectively, after gradual acclimatization (over 15 min).

To assess whether covering the fish larvae with agarose affects the oxygen concentration available to it, prior to the three randomly chosen experiments, we compared the oxygen concentration in the E3 medium and inside the glass capillary filled with agarose and submerged in E3 medium. Both measurements were conducted in each of the three experimental temperatures. To assess whether temperature affected the oxygen concentration inside the sample chamber of the Lightsheet Z.1 microscope during the experiments, we measured the oxygen concentration in the E3 medium inside the sample chamber for the three randomly chosen experiments at each of the three temperatures. All of the measurements of oxygen concentration were performed using a miniature UNISENSE oxygen probe (model OP-MR-706808).

### Data analysis

Time lapsed images were analysed using Zeiss ZEN software. The region of interest (ROI) was selected in every recorded time-lapsed image, in a single region of the OT, where the first incoming signal in response to the pixel movement was detected based on the F_max_/F_base_ factor. This single region was constantly monitored throughout the duration of pixel presentation. The mean ratio was equal to 1.251, 1.154 and 1.152 at 18, 23 and 28°C, respectively. The fluorescence peak was treated as a sign of spotting the moving target by the zebrafish, the first neural cell or cells that lit up due to the flow-through of Ca^2+^ (Fig. 3A), and we marked the ROI in this area. The ROI had the shape of a 10 µm diameter ring. As a result, the relative fluorescence intensity for a given time-point was obtained (Fig. 3A). All further analyses were performed in Python ver. 2.7 (Open Source, download: https://docs.python.org/2/) and MatLab (MathWorks) ver. R2018b. First, the peaks of relative florescence were identified for each graph. We used the ‘*findpeaks*’ algorithm from MatLab to identify the peaks and to find the location of the maxima; the beginning and end of each peak, and to measure the basal and maximal fluorescence of the peaks in each of the graphs using Python. The basal fluorescence was assessed by measuring the basal fluorescence level between the peaks (Fig. 4B). In some graphs, double tops on the peaks occurred. Only the first five peaks were considered for further analysis. Peaks that were the result of an artefact were excluded from the analysis. In total, we analysed 30 peaks for 18, 40 for 23 and 30 for 28°C.

The detection distance, assumed as the maximal distance that triggers the first neural activity in a region of the OT in response to the visual stimuli, was determined using the Pythagorean theorem. The triangle was created by determining the relative spatial location of the screen surface, the eye of the fish and the position of the pixel on the screen that triggered the first observed neural activity in a region of the OT. The length |bc| (Fig. 2B) linked the distance between the screen surface and the eye of the fish (Fig. 2A,B). The length |ab| linked the position of the pixel on the screen triggering the first observed neural activity in a region of the OT and the point on the screen opposite the eye of the fish (Fig. 2B); these two lengths created the right angle (90°). The third side of the triangle, assumed to be the detection distance – |ac|, was designated by substituting data from the length of the other two sides to the Pythagorean theorem (in our case: |ab|^2^+|bc|^2^=|ac|^2^). We converted the movement speed of the pixel on the screen (2.1 mm s^−1^) to the distance travelled by the pixel on the |ac| section.

The mean duration of peaks was calculated as the difference in time between the beginning and end of a peak (Fig. 4B). To calculate the FWHM of each peak, we looked for the width on the fluorescence graph measured between those points on the Y-axis, which is half of the maximum amplitude (Fig. 4B). The mean time between adjacent peaks was calculated as the mean difference in time between the end of one peak and the beginning of the adjacent peak (Fig. 4B).

All statistical analyses were performed using IBM SPSS Statistics 25.0 and TIBCO Statistica 13.3. The *P*-levels below 0.05 were deemed statistically significant. For all variables, their normal distribution fit and skewness were checked at each temperature and Levene's test for the homogeneity of variances was performed. We noted no significant skew (absolute value of skewness<2) for the variables, with the exception of the time between adjacent peaks. Therefore, the one-way analysis of variance (ANOVA) (with the possible extension using the Brown-Forsythe test) was performed to compare the detection distance, duration of peaks and FWHM. The post-hoc comparisons were performed using Tamhane's test for the data with even variances, and Scheffe's test for the data with uneven variances. The Kruskal–Wallis ANOVA was used to compare the time between two adjacent peaks in each temperature with the post-hoc Dunn's test.

## Supplementary Material

Supplementary information
