# Supplementary material for: The effects of temperature on the proxies of visual detection of Danio rerio larvae: observations from the optic tectum
Source: Biol Open. 2020 Jul 21;9(7):bio047779. doi: 10.1242/bio.047779 (PMC7390641; doi:10.1242/bio.047779)
Supplement: Supplementary information [file biolopen-9-047779-s1.pdf]

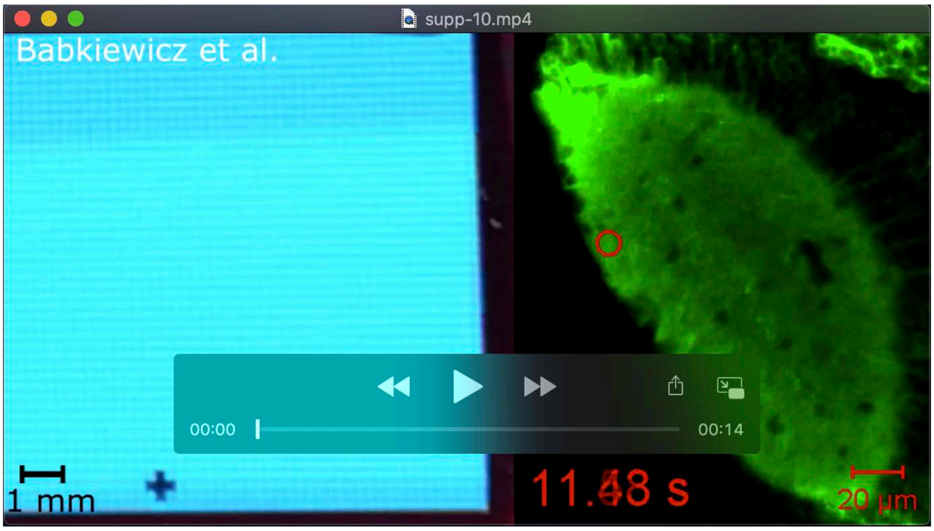

Movie 1

Synchronization of the signal in the *OT* with the movement of the pixel

On the left there is shown a pixel movement displayed on the OLED screen inside sample chamber of the Lightsheet Z.1 microscope and on the right there is shown the signal in the *OT* visually synchronized with the movement of the pixel.

Biology Open • Supplementary information

Supplementary Figures

Graphs containing the baseline traces of fluorescence (in relative units) in examined zebrafish larvae in each of the three experiemntal temperatures, recorded to screen the *OT* region for any spontaneous and basal fluorescence. The grey dashed line indicates the time-point when the stimulus onset occurred.

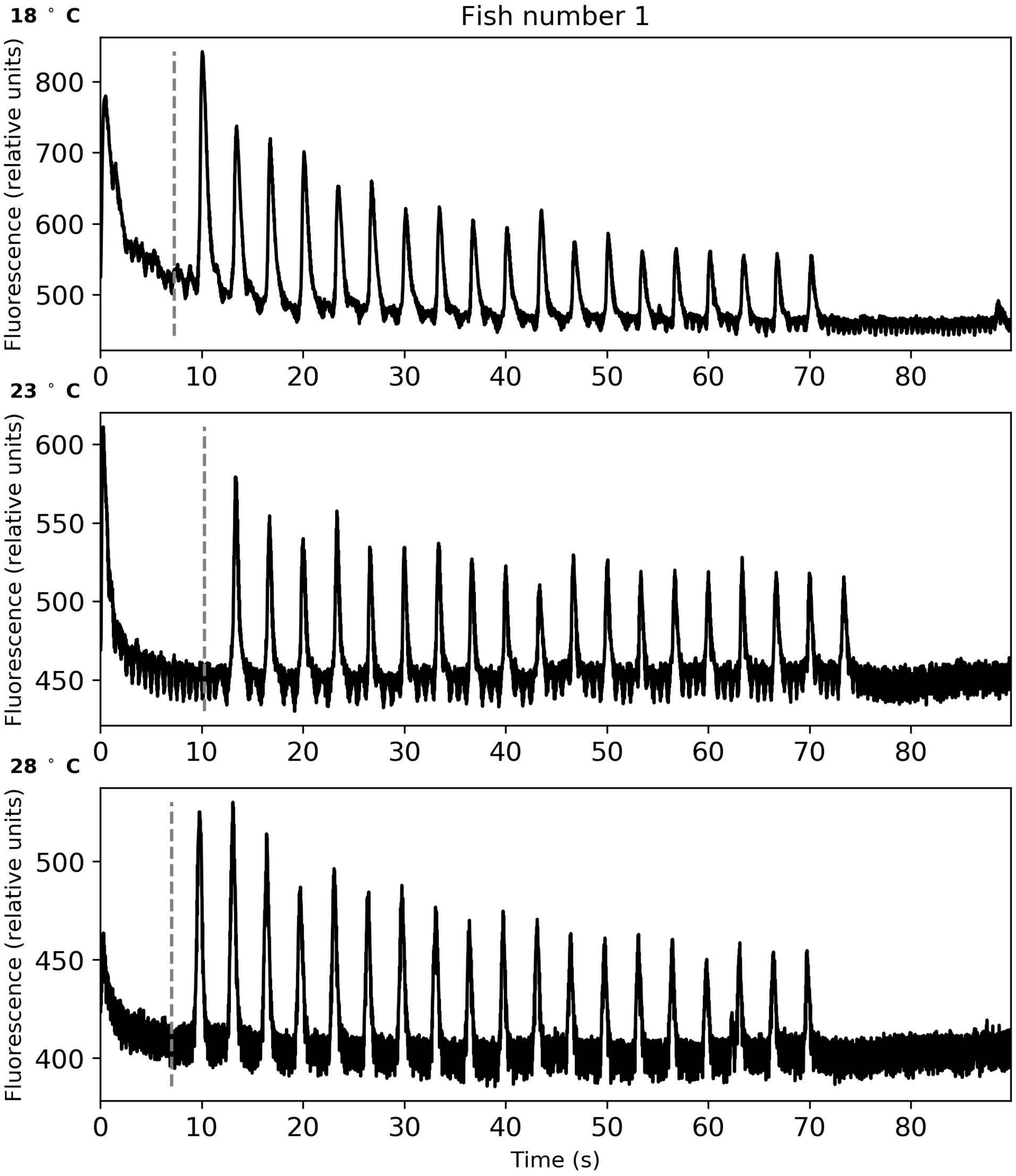

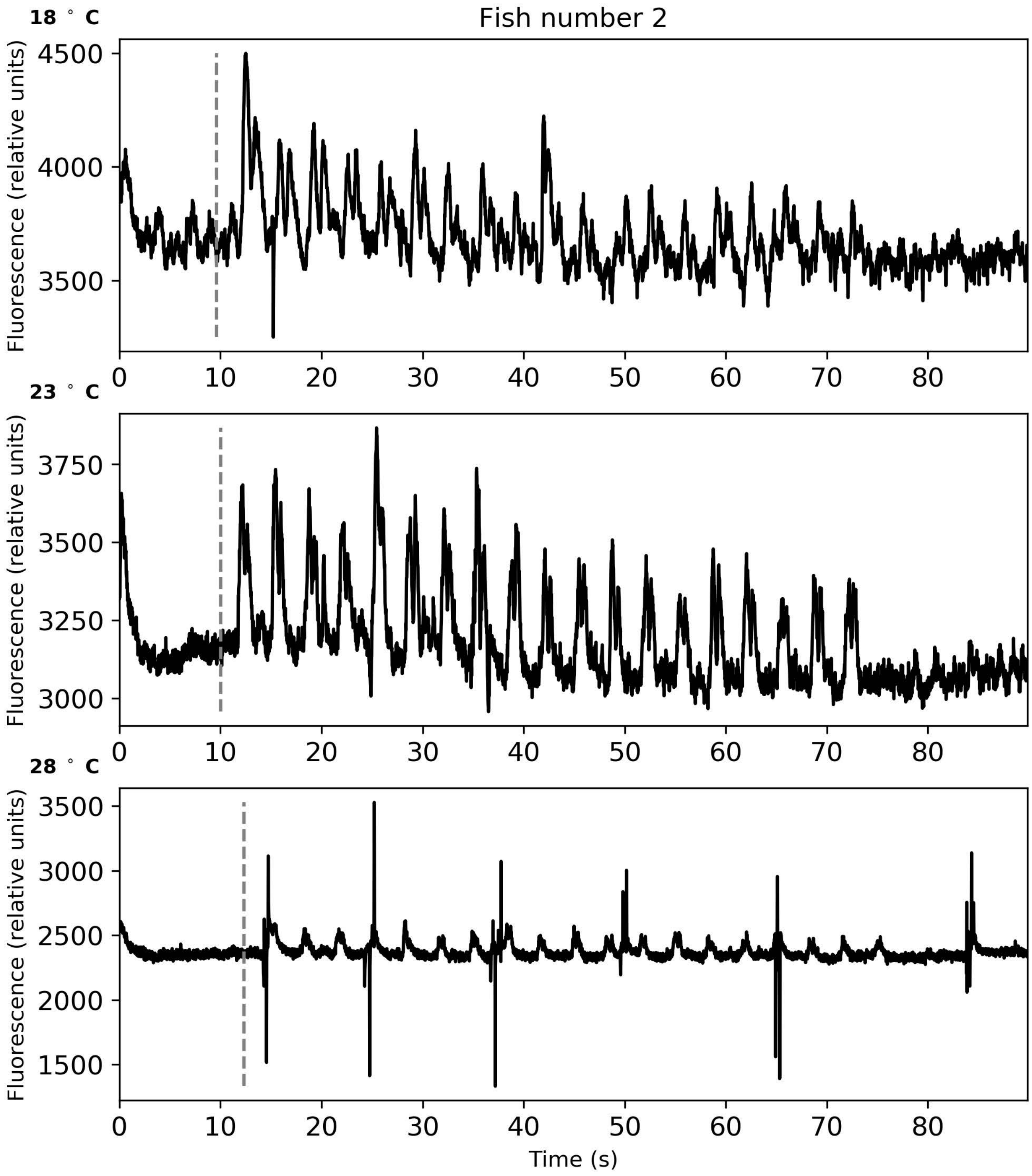

Biology Open • Supplementary information

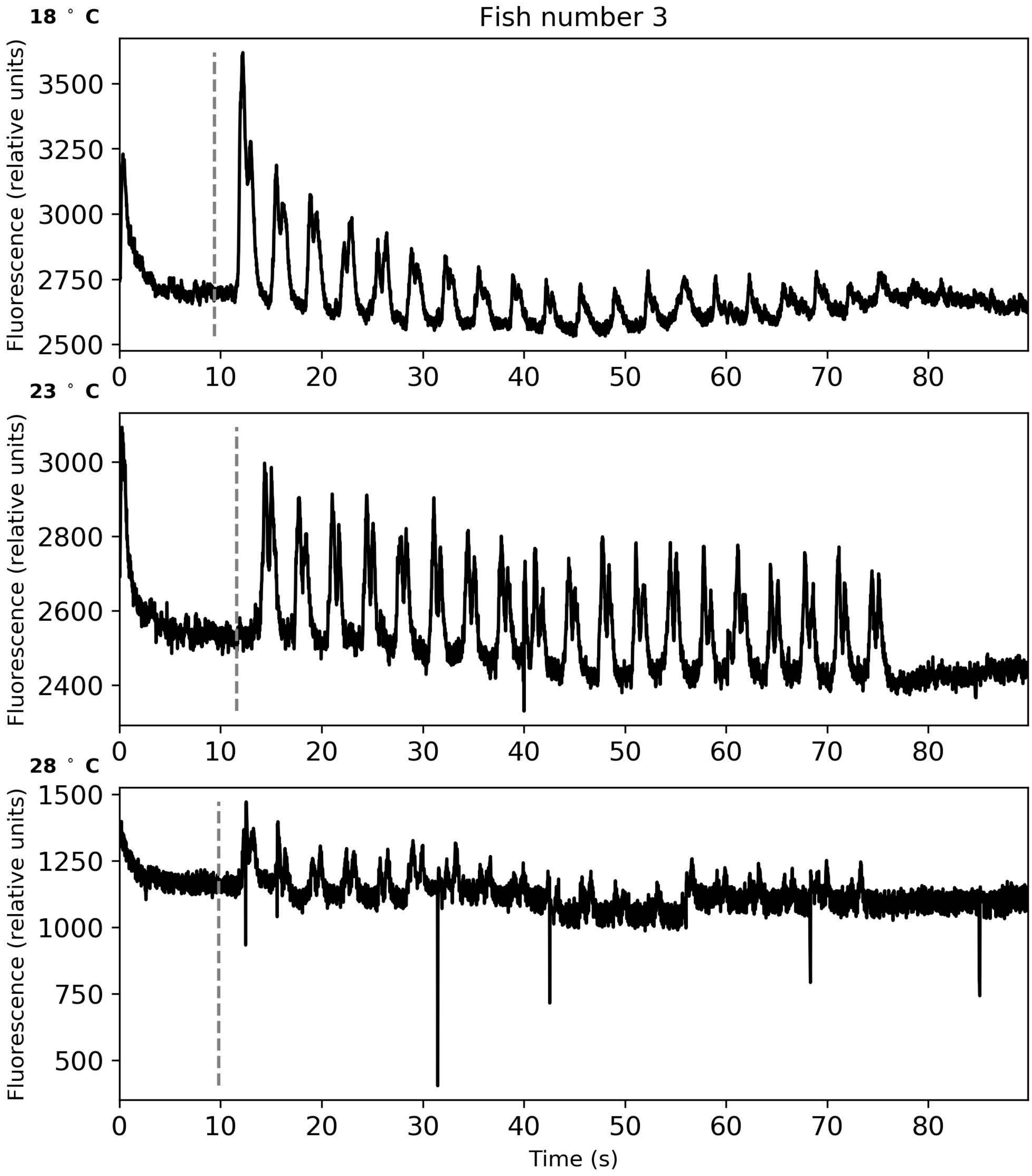

Biology Open • Supplementary information

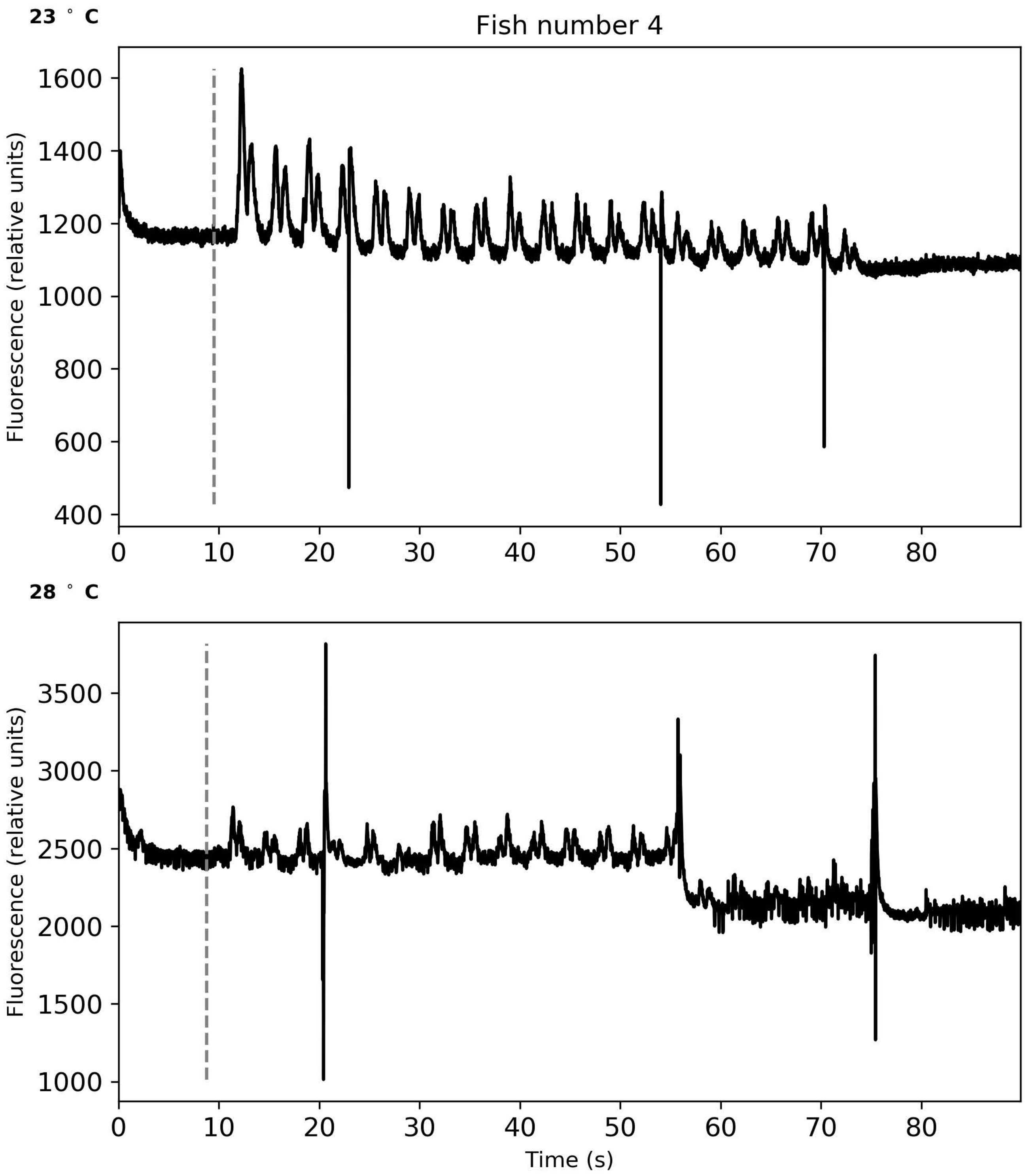

Biology Open • Supplementary information

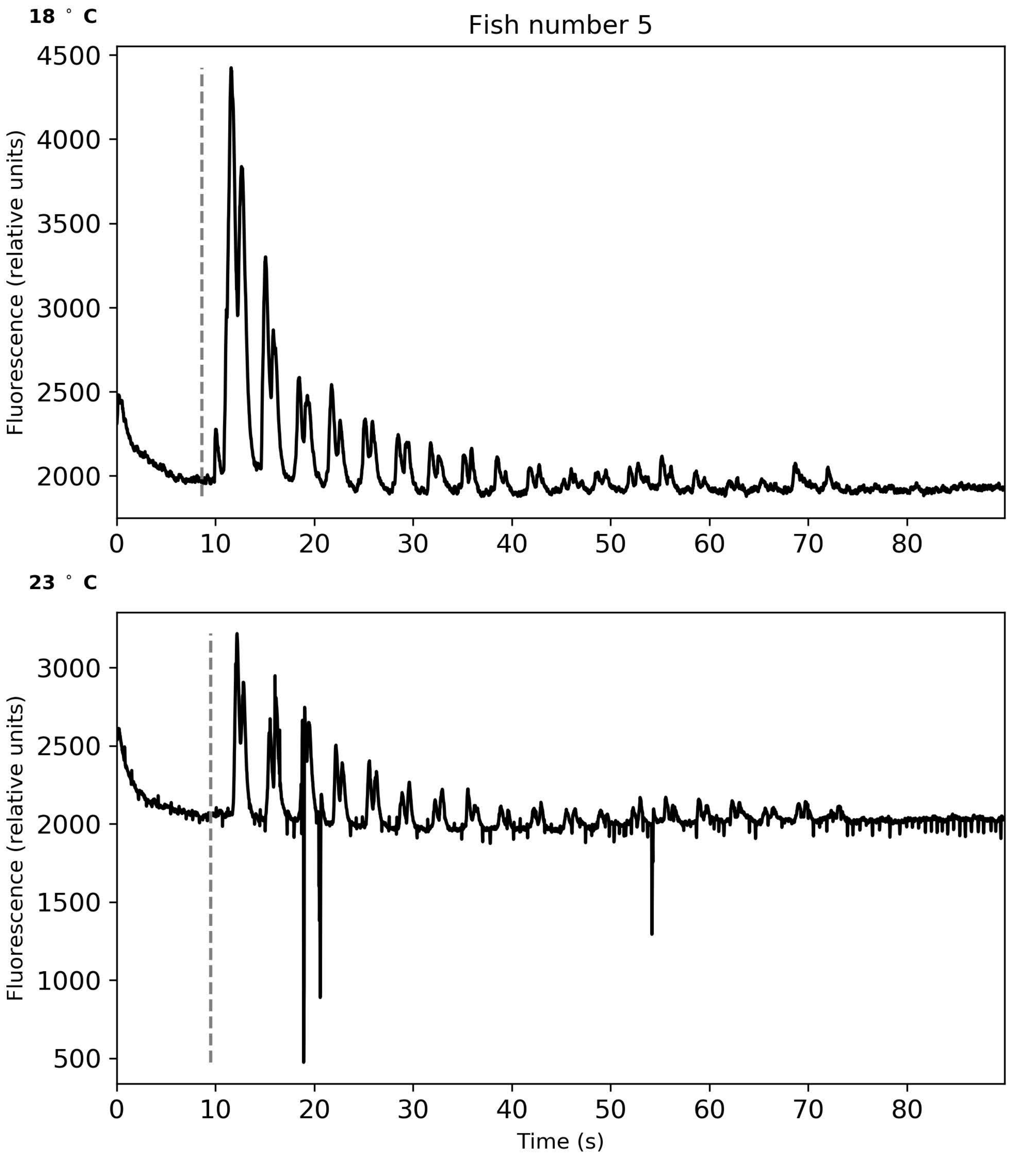

Biology Open • Supplementary information

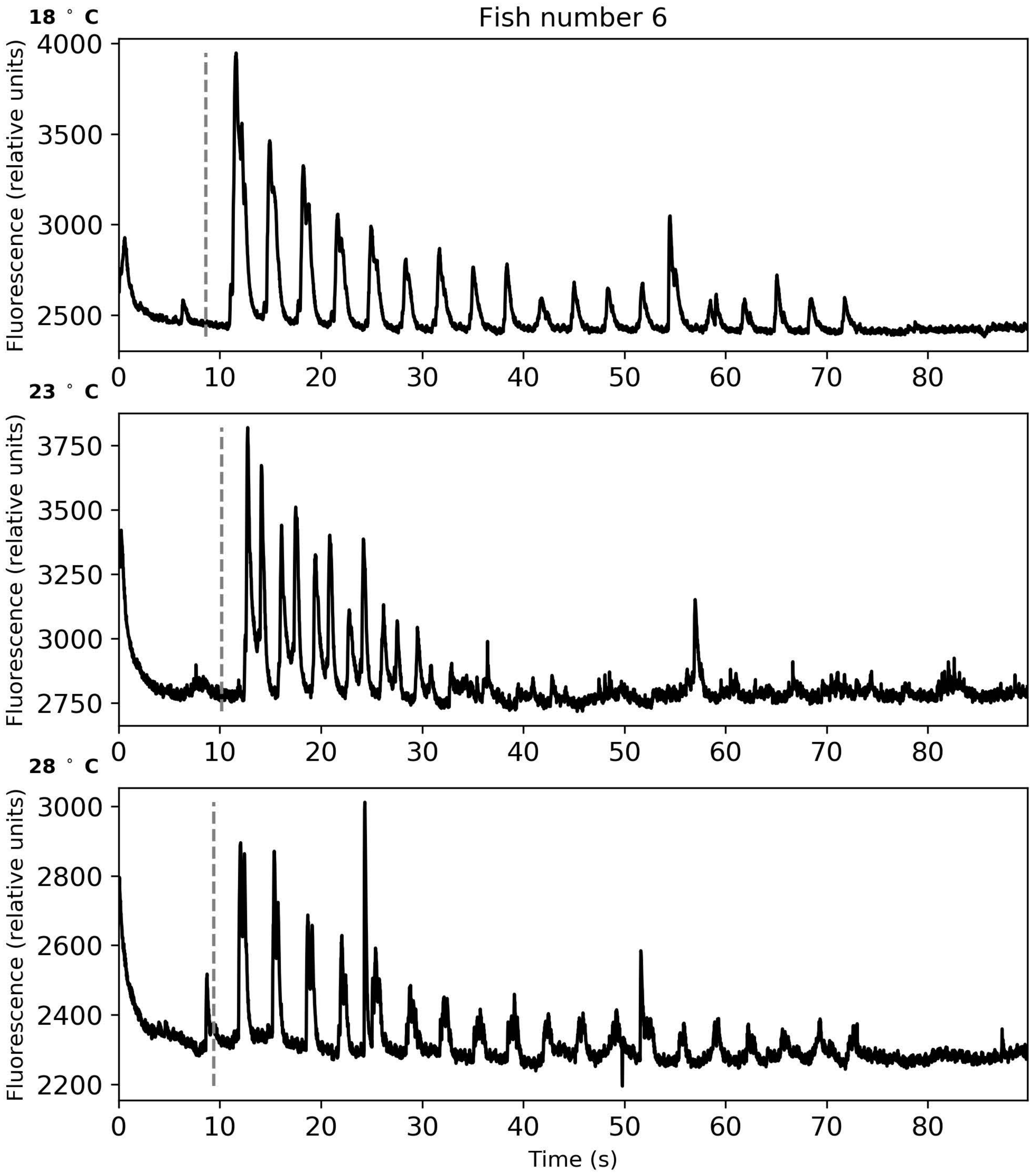

Biology Open • Supplementary information

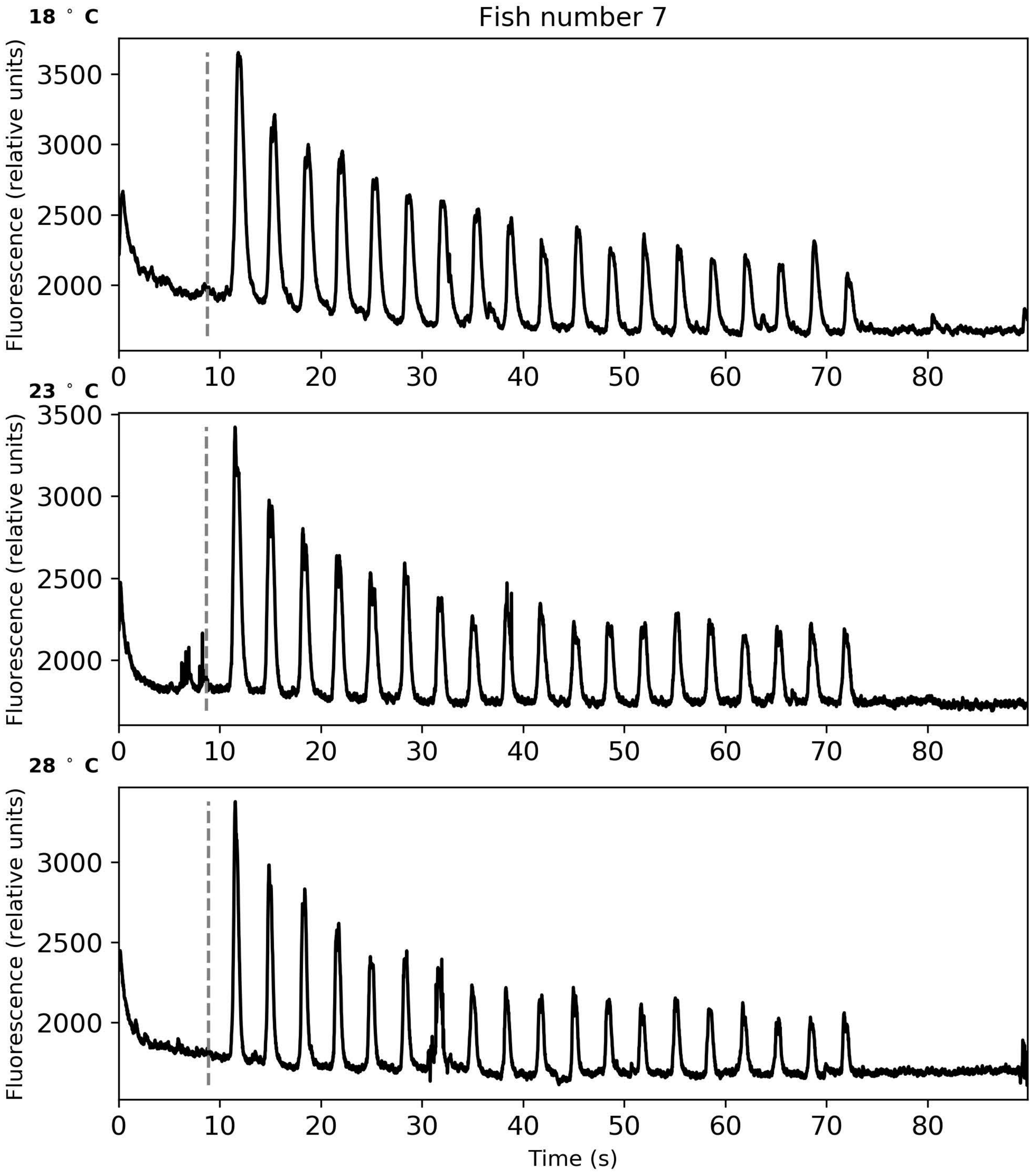

Biology Open • Supplementary information
